# The Effect of Periarticular Injection of Methylprednisolone Acetate in Patients with Primary Osteoarthritis of the Proximal Interphalangeal Joints: A Case Controlled Study

**DOI:** 10.1155/2018/7561209

**Published:** 2018-11-14

**Authors:** George Habib, Fahed Sakas, Suheil Artul, Fadi Khazin

**Affiliations:** ^1^Rheumatology Unit, Laniado Medical Center, Faculty of Medicine, Technion, Israel Institute of Technology Haifa, and Rheumatology Clinic, Nazareth Hospital, Nazareth, Israel; ^2^Department of Family Medicine, Rambam Medical Center, Haifa, Israel; ^3^Radiology Department, Nazareth Hospital, Nazareth, Israel; ^4^Department of Orthopedics, Carmel Medical Center, Haifa, Israel

## Abstract

**Backgrounds:**

Primary osteoarthritis of the proximal interphalangeal joints (PIPJ) is a common entity. It could be associated with local pain that has no effective treatment. Local subcutaneous periarticular injection of methylprednisolone acetate (MPA) was evaluated in a prospective case-control study.

**Methods:**

Patients with painful osteoarthritis of the PIPJ for more than 1 month not responding to nonsteroidal meds were prospectively recruited. Radiographic, demographic, clinical, and lab parameters were documented. Visual analogue scale (VAS) was documented regarding the level of PIPJ pain prior to the injection. Patients had local subcutaneous periarticular injection at the medial and lateral sides of each painful PIPJ of one hand, of 8 mg (0.2 ml) of MPA mixed with 0.1 ml of lidocaine 1% (group 1) at each side. Age- and sex-matched control group were given 0.3 ml of normal saline using the same approach (group 2) at each side. VAS was evaluated 1, 4, and 10 weeks following the injection and compared to baseline levels using Wilcoxon's ranks signed test.

**Results:**

Eighteen and sixteen patients were recruited in group 1 and group 2, respectively. There were 11 females in group 1 with mean age of 52.7 ± 9.2 years. Mean VAS in group 1 at baseline was 67 and at weeks 1, 4, and 10 was 23 (p=0.001), 29 (p=0.001), and 55 (p=0.043), respectively. Mean VAS in group 2 at baseline was 65 and at weeks 1, 4, and 10 was 43 (p=0.005), 64 (p=0.534), and 69 (0.698), respectively.

**Conclusions:**

Subcutaneous periarticular injection of MPA + lidocaine at the PIP joints resulted in a small but significant improvement that gradually diminished with time across the week 10, among patients with primary OA of hands.

## 1. Introduction

Primary osteoarthritis of the hands is a common clinical entity [1]. It usually affects trapeziometacarpal joint and proximal and/or distal interphalangeal joints (PIPJ and DIPJ) [2]. Its incidence increases with age and its prevalence ranges from 27 to 80% among people older than 45 years, where women are more affected than men [3]. There is also a strong genetic predisposition [4]. Other risk factors for the development of primary OA of hands include moderate alcohol consumption and probably hyperlipidemia [5, 6]. Bone mineral density was found to be inversely related [7]. There are inconsistent findings regarding body mass index and smoking [5, 8]. Endothelin-1 and adipokine may have a role in the pathogenesis of primary OA of the hands [9, 10]. The degenerative changes consist of new bone formation, joint space narrowing, subchondral cysts, and/subchondral sclerosis [11]. Bone erosion could also be seen. These changes can result in joint area swelling, stiffness, pain, deformity, and reduced function [12]. Currently there is no cure for this entity and patients usually see their primary practitioners due to either deformity, pain, or reduced hand function.

Pain can be approached by different modalities of treatment including topical and/or systemic nonsteroidal anti-inflammatory drugs (NSAID), simple analgesics, warm paraffin paths, physical therapy, surgery, alternative therapy, and/or local corticosteroid injection [13, 14] The local steroid injection is usually directed intra-articularly. Due to the small size of the PIPJ, attempting an intra-articular approach there could be very painful and intolerable by the patients.

There are no studies in the literature about the efficacy of periarticular corticosteroid injection at the PIPJ area, on pain relief among patients with primary OA of the PIPJ. In this controlled study we evaluated the effect on pain relief of local subcutaneous periarticular methylprednisolone acetate (MPA) versus saline injected at both medial and lateral sides of the PIPJ.

## 2. Methods

Nonselected patients attending the rheumatology clinic at our hospital, with painful primary osteoarthritis of the PIPJ, were asked to participate in our study. The diagnosis of primary OA of the PIPJs was done based on the Chingford Study [15]. After consent, demographic, clinical, and laboratory parameters of the patients in addition to pain grading of the PIPJ were documented. The evaluation of pain was done using visual analogue scale (VAS), from 0 (no pain) to 100 (worse pain ever experienced).

Following that, patients had periarticular injection of the painful PIP joints of one hand (the one that had more pain or nondominant hand if both hands had similar levels of pain). The injections were done using 1 ml syringe with 29/30 G built in-needle (undetachable needle) (BD Micro-Fine, USA) and inserted subcutaneously at an angle of about 45° at the most medial and most lateral parts of the PIPJ line, after producing a skin fold by the other hand. Prior to that the skin was cleaned by chlorhexidine 70% alcohol. At each side a 8 mg (0.2 ml) of methylprednisolone acetate (MPA) (Pfizer N/V, Belgium) mixed with 0.1 ml of lidocaine 1% (Teva, Petach Tekva, Israel) was injected (group 1) [16]. Joint pain evaluations were repeated 1, 4, and 10 weeks at the same clinic following the local injection. Age- and sex-matched group of patients were recruited also from the same clinic as a control group (group 2) using 0.3 ml of normal saline utilizing the same approach and methods of evaluation prior to and following local saline injection.

Exclusion criteria included patients with inflammatory presentation and evidence of synovitis from systemic diseases like rheumatoid arthritis and psoriatic arthritis, patients with erosive osteoarthritis, patients with symptom duration of less than 1 month, patients with a previous injection at or around the PIP joint during the last 3 months, patients with evidence of skin infection at the PIP area, patients unable to sign a consent form, and patients unable to come for follow-up. Patients on anticoagulants were not excluded.

Mann-Whitney and Chi square tests were used to compare between the continuous and categorical parameters of the two groups at baseline, using 21 version SPSS software. Wilcoxon's ranks signed test was used to compare between the VAS scores of the patients at each time point with the VAS scores at baseline in each group.

This study was approved by the local ethics committee at the Laniado Hospital, and all the patients signed a consent form.

## 3. Results

Eighteen and sixteen patients were recruited in group 1 and group 2, respectively, during 1 year. Demographic and clinical parameters of the two groups are shown in Table 1. Both groups were comparable. No patient withdrew from further planned injections after starting it due to pain.

Table 2 summarized the VAS of all the patients at different time points and in both group 1 and group 2, respectively. Significant improvement was seen at all-time points in group 1 and at week 1 only in group 2.

One patient developed hypopigmentation of the skin at the area of an injection 3 months later, with the size of 3x4 mm. No patient developed continuing or serious local bleeding.

## 4. Discussion

The results of our study show that periarticular subcutaneous injection at the PIPJ results in a significant improvement in joint area pain. This significant improvement lasts for at least 10 weeks. This indicates that the infiltrated periarticular structures had an important role in the symptoms of the painful osteoarthritic PIPJ or that at least some of the infiltrated material finds its way in to the joint space resulting in the observed improvement. Combination of both options could also be a possibility here.

Diminishing favorable effect of local corticosteroid injection is usually the rule, especially among joints or areas with chronic degenerative changes that are subject to continuous physical activity [17].

Normally, underneath the skin and subcutaneous tissue at the lateral and medial parts of the PIPJ, lies the joint capsule, composed of the different ligaments that cover the synovial membrane of the PIPJ. In PIPJ osteoarthritis, bony prominences and cysts develop, with or without sclerosis. Element of synovitis could also be seen [18]. There is no data in the literature about ligament involvement at the osteoarthritic PIPJ area. These ligaments may also have a role in PIPJ area pain and function, and corticosteroids plus lidocaine injection around these layers might affect pain stimulating changes.

Technically the procedure is simple, very tolerable by the patients, and no special skills are needed to penetrate the skin at the most medial and lateral parts of PIPJ line. So after observing the procedure only one time, physicians could be competent in performing this procedure. Periarticular subcutaneous injection using our method is supposed to be less traumatic exposing the patients to lower risk of serious or continuing bleeding even among those on anticoagulants.

It is not clear weather injecting one side at the PIP joint would have the same clinical effect as injecting both sides as we did. Although we mentioned that some material would spread around, we are not sure that enough injected material would get to the other side of the joint. Further studies of utilizing injections at one side versus both sides are needed.

The issue of repeated injections is still not clear using our method. It is accepted not to inject intra-articularly less often than 3 months. It might be that the same rule could be applied here also.

The advantage of the type of syringe we used that is the needle is undetachable and no fear of splashing material is faced by the physician once any unexpected resistance is encountered during the injection. Yet good common practice requires changing the needle for the sake of sterility. However if the rules of sterility are well kept, this issue could be overcome.

Ideally, using steroids alone, comparing it with lidocaine in a second group and sterile water in a third group, would define more clearly the role of either compound (steroids versus lidocaine) in the observed improvement of pain in our study. However, since in common practice both steroids and lidocaine are used together we thought as a first step to use both compounds together, and once a significant improvement had been documented, future studies could be done comparing the efficacy of each compound alone. Also, patients usually complain of sore feeling at the injected area when steroids alone are used for local injection, and the premixing with lidocaine prevents this feeling (personal experience of the first author).

Hypopigmentation is an uncommon adverse effect of local corticosteroid injection [19]. The estimated prevalence is about 3% and up to 11% in some studies.

There are some drawbacks regarding our study; we did not evaluate the impact of the local injections on finger or hand function, although many patients in group 1 reported improvement in finger and hand function. Planned future studies hopefully will address this issue.

Better strength of the results could be achieved by blinding, randomization, and/or increasing the number of patients. We think that the strength of evidence of our work as a case-control study using a well-accepted end point evaluated by the VAS is very reasonable. Also, we were not sure initially that enough number of candidates could be recruited to the study, in order to guarantee a good randomization. Regarding patients blinding at least, both groups of patients were told that the type of injection they were getting was supposed to relief pain.

In conclusion, our study showed a small but significant difference from the MPA corticosteroid/lidocaine intervention that gradually diminished with time across the 10-week follow-up period. Both patients and clinicians would need to understand the length of benefit which might be expected when discussing this procedure as a potential management option, as well benefits and any potential harm.

## Figures and Tables

**Table 1:** Demographic, clinical, and radiographic parameters of the patients.

**Parameter**	**Group 1**	**Group 2**	**P value**
(i) F:M	11:7	10:6	0.939
(ii) Age*∗*	52.75±9.2, 47-76	57.5±8.45-70	0.118
(iii) Duration of pain*∗*	4.2±3.2, 1-12	3.18±2.5, 0.5-10	0.294
(iv) No. of PIPJ injected*∗*	2.83, 0.98, 1-5	2.5, 0.96, 1-5	0.824
(v) VAS*∗*	64.58±10.54, 45-88	69.8±10.8, 54-90	0.151
(vi) C-RP*∗*	2.89±2.03, 0.1-6.3	2.25±2.0, 0.1-8	0.347
(vii) ESR*∗*	15.5±7.55, 7-33	16.4±10.1, 5-41	0.714

**∗**Mean± SD, range.

F=female, M=male, PIPJ=proximal interphalangeal joint, VAS= visual analogue scale, ROM=range of motion, C-RP= C-reactive protein, and ESR= erythrocyte sedimentation rate.

**Table 2:** VAS score at different time points among group 1 and group 2 patients.

	G r o u p 1					G r o u p 2			
Patient	VAS at baseline	VAS at W1	VAS at W4	VAS at W10	Patient	VAS at baseline	VAS at W1	VAS at W4	VAS at W10
1	55	22	18	51	1	68	24	56	72
2	58	13	19	61	2	47	56	62	66
3	61	5	NA	75	3	65	42	48	48
4	72	15	16	22	4	87	35	66	82
5	76	31	25	43	5	92	42	90	86
6	66	11	38	44	6	95	56	88	76
7	88	9	16	65	7	56	65	63	68
8	64	NA	48	72	8	57	68	NA	88
9	71	8	NA	21	9	84	22	75	76
10	65	44	39	51	10	74	10	88	NA
11	68	27	62	54	11	46	56	67	72
12	56	18	15	NA	12	56	42	22	40
13	64	42	39	59	13	65	34	NA	56
14	71	32	24	66	14	68	22	82	NA
15	85	24	43	52	15	47	56	62	74
16	53	NA	34	67	16	82	92	65	62
17	65	24	74	NA					
18	74	10	NA	80					

**Mean VAS**	67	23	29	55		68	45	67	69

**P value**		0.001*∗*	0.001*∗*	0.043*∗*			0.005*∗*	0.534*∗*	0.698*∗*

**∗**Compared to baseline in the same group.

VAS=visual analogue scale, W1=1 week following the periarticular injection, W4= 4 weeks following the periarticular injection, W10=10 weeks following the periarticular injection, and NA=not available.

## Data Availability

The data used to support the findings of this study are available from the corresponding author upon request.

## Supplementary Materials

Supplementary Materials Figure 1: subcutaneous periarticular injection at the medial part of the 4th PIP joint.
