# Supplementary figures and images for: The Effect of Periarticular Injection of Methylprednisolone Acetate in Patients with Primary Osteoarthritis of the Proximal Interphalangeal Joints: A Case Controlled Study
Source: Pain Res Treat. 2018 Nov 14;2018:7561209. doi: 10.1155/2018/7561209 (PMC6261070; doi:10.1155/2018/7561209)

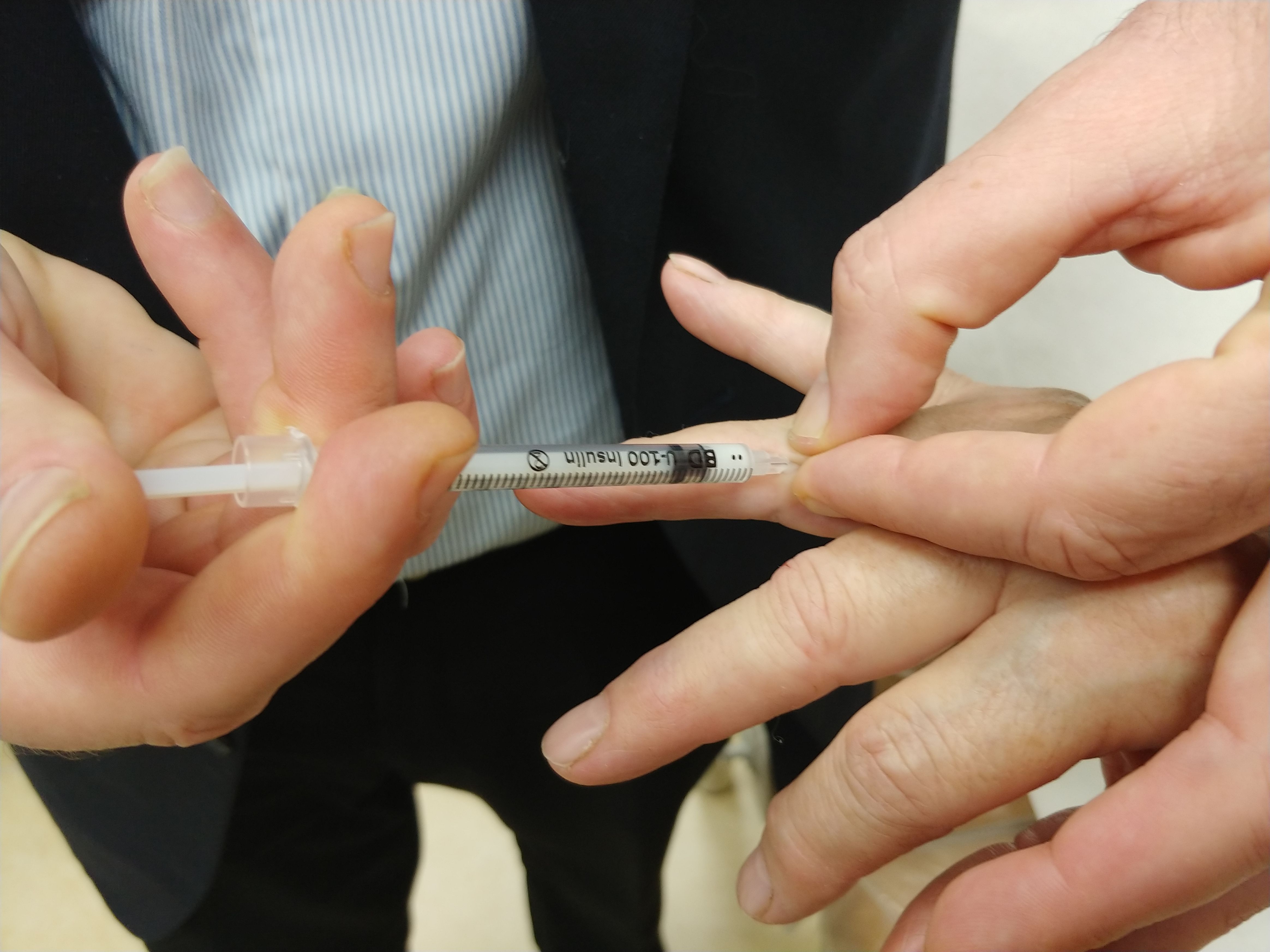

Supplement: Supplementary Materials — Figure 1: subcutaneous periarticular injection at the medial part of the 4th PIP joint. [file 7561209.f1.jpg]
